# A Novel Approach to Interrogating Whole Genome Sequencing Data to Optimise Clinical Utility

**DOI:** 10.1002/mgg3.70248

**Published:** 2026-06-11

**Authors:** Sarah Sonner, Caoimhe McKenna, Shirley Heggarty, Cassie Hamill, Cheryl Flanagan, Shane McKee, Katie Kerr, Rasha Alhazzaa, Amy Jayne McKnight, Fionnuala Mone

**Affiliations:** ^1^ Centre for Public Health Queen's University Belfast Belfast UK; ^2^ Northern Ireland Genomic Medicine Centre Belfast Health and Social Care Trust Belfast UK; ^3^ Regional Molecular Diagnostics Service (Germline) Belfast Health and Social Care Trust Belfast UK; ^4^ Health Informatics Department Saudi Electronic University, College of Health Sciences Dammam Saudi Arabia; ^5^ Centre for Fetal Medicine Belfast Health and Social Care Trust Belfast UK

## Abstract

**Objective:**

To evaluate the performance of the genomic analytics applied to the Northern Ireland cohort recruited to the 100,000 Genomes Project, focusing on diagnostic yields in first‐line analysis and revisiting inconclusive cases with rare diseases.

**Methods:**

Whole genome sequencing (WGS) and initial data processing were performed centrally by Genomics England (GEL), consistent with a national protocol. A novel internal approach to variant selection and interpretation was designed using a two‐pronged pathway; with initial prioritisation using GEL virtual panels and subsequent Exomiser‐mediated reanalysis of remaining undiagnosed cases. Retrospective collection of genomic records was completed for service evaluation.

**Results:**

Of 440 patients recruited 20.2% (*n* = 89/440) received a monogenic diagnosis; 69.7% (*n* = 62/89) via the initial interpretation pathway (analysed between 2019 and 2021) and 30.3% (*n* = 27/89) via the secondary pathway (analysed between 2019 and 2025). The majority of variants missed by the initial analysis were because the diagnostic gene was not included as a target on the applied panel(s) at the time of analysis. The median turnaround time for the initial approach was 644.5 days (range: 315–1342) and for the secondary approach was 1766.5 days (range: 862–2311). The secondary approach was found to be more effective in prioritising pathogenic variants; 69.7% (*n* = 62/89) versus 89.9% (*n* = 80/89) *p* = 0.0008.

**Conclusion:**

Pre‐classification variant selection and reanalysis of undiagnosed cases are critical to optimise quality improvement in the clinical delivery of WGS. Variant selection via virtual panels can expedite interpretation but may miss clinically relevant findings, leaving cases undiagnosed. Revisiting these cases gave an additional ~6% yield. For efficient variant selection in both initial and follow‐up analysis, a multi‐disciplinary approach is required.

## Graphical abstract

Pre‐classification variant selection and reanalysis of undiagnosed cases are critical to optimise the quality improvement in the clinical delivery of whole genome sequencing in rare disease. Variant selection via virtual panels can expedite interpretation but may miss clinically relevant findings, leaving cases undiagnosed for rare disease. Revisiting undiagnosed rare disease cases provided an additional ~6% yield.

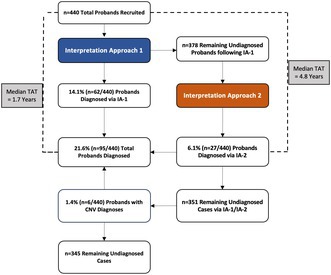

## 1 Introduction

With over 8000 known conditions, establishing a rare disease (RD) diagnosis is complex, causing many patients and families to endure a prolonged ‘diagnostic odyssey’ seeking an explanation for their symptoms (Nguengang Wakap et al. 2020). Approximately 80% of RDs are caused by pathogenic single gene variation, making Next Generation Sequencing (NGS) instrumental in odyssey reduction (Wu et al. 2020). Clinical use of whole genome sequencing (WGS) enables massively parallel sequencing of multiple genes, increasing the likelihood of detecting a pathogenic variant with a single test (Fernandez‐Marmiesse et al. 2018).

Healthy human genomes may show variation at up to 5 million sites compared to the human reference genome (Bagger et al. 2024; 1000 Genomes Project Consortium et al. 2015). The difficulty lies in determining which variation, if any, is causative of a patient's phenotype. To alleviate interpretational burdens, bioinformatic pipelines are employed to prioritise and flag variants deemed most likely to be causative (Roy et al. 2018). However, diagnosis is not guaranteed. A patient's condition may not have a genetic origin, or causative variants can be filtered out depending on how the pipeline is constructed and what it prioritises. Moreover, some causative variants, especially novel ones, may lack sufficient evidence of pathogenicity to be deemed diagnostic and therefore cannot be flagged by bioinformatic pipelines (Record and Reilly 2024). As the data generated from sequencing can be stored long‐term, these inconclusive cases can be revisited with additional clinical or research evidence and may be diagnosed without additional sample from the patient (Robertson et al. 2022; Deignan et al. 2019). However, this generates further interpretational burdens for staff.

The Northern Ireland Regional Molecular Diagnostics Service (NIRMDS), with academic support from Queen's University Belfast (QUB), was a regional hub for the 100,000 Genomes Project by Genomics England (GEL), which performed WGS on ~100,000 people under the RD or cancer arms. Patients living with an undiagnosed RD were recruited, aiming to establish a molecular diagnosis (Samuel and Farsides 2017; Kerr et al. 2022). Although regional centres primarily adhered to national protocol for sequencing and data handling, there was scope to tailor the specific analysis approach. NIRMDS developed a novel two‐step workflow. First, a review of all variants flagged by GEL pipelines. And second, a follow‐up of the remaining undiagnosed patients, analysing all detected variants using Exomiser (Smedley et al. 2015) with enhanced phenotypic review.

This study aimed to evaluate NIRMDS performance in diagnosing 100,000 genomes project RD patients through the following objectives: (i) assess internal performance metrics, that is, diagnostic yield of monogenic diseases and turnaround times (TAT), overall and per individual pathway; (ii) determine plausible explanations for diagnoses being missed by first analysis and found in the secondary; and (iii) establish the diagnostic potential of both approaches.

## 2 Methods

### 2.1 100,000 Genomes Project

The methodology for the 100,000 Genomes Project has previously been published (Caulfield et al. 2017). Briefly, national recruitment occurred between 2015 and 2018 (Stafford‐Smith et al. 2025). Sequencing and initial data processing were performed centrally by GEL, and regional centres were responsible for variant interpretation and reporting for their patients. Remote local access to variant data was facilitated by digital health company Congenica, which produces clinical decision supports for interpreting and classifying NGS detected variants (Congenica, n.d.). The Congenica bronze service was employed as a NIRMDS interface to access filtered variant data on the GEL interpretation portal.

Initial interpretation was standardised across all regional centres, including NIRMDS, using a panel‐based approach to filter and rank variants in tiers (Genomics England 2025). Phenotypic information was provided by referring clinicians as Human Phenotype Ontology (HPO) (Robinson et al. 2008) terms, which enabled the application of virtual gene panels via PanelApp (Martin et al. 2019), a publicly available crowdsourcing tool which utilises input from the global scientific community. Within these panels, genes are colour‐coded using a traffic light system to demonstrate the level of available evidence supporting gene‐disease association (Figure S1) and can change to reflect newly publishedevidence (Leigh et al. 2025). Only variants detected within ‘Green Genes’, progressed along the bioinformatic pipeline to be ranked into one of four tiers based on molecular impact (Table S1). At the initial analysis stage, only Tier 1 and 2 variants, the most likely to be causative, were released to regional centres via the interpretation portal for analysis. From these, variants of interest (VOIs) were selected as possible causes of patient phenotypes. Deeming a VOI as diagnostic required formal classification as per American College of Medical Genetics and Genomics (ACMG) and Association for Clinical Genomic Science (ACGS) criteria (Richards et al. 2015; Masson et al. 2022; Durkie et al. 2024).

### 2.2 NIRMDS Workflow

The NIRMDS developed a novel approach for VOI selection, which are brought forward for formal classification. This was a two‐step pathway comprised of an initial interpretation approach (IA‐1) and a secondary follow‐up interpretation approach (IA‐2), allowing any undiagnosed cases to be tactically revisited.

All cases were initially investigated via IA‐1, following the release of Tier 1 and 2 variants. After clinical review, referring clinicians selected a maximum of two VOIs per patient which they considered consistent with the patient phenotype they observed. This relied heavily on the clinician's experience and knowledge of dysmorphology and gene‐disease relationships (Reardon and Donnai 2007).

Formal classification of a variant required a consensus to be reached through a multi‐disciplinary team (MDT) discussion, led by clinical scientists and validated with Sanger sequencing (Sanger et al. 1977). Positive/diagnostic findings were collated into formal reports and disseminated to referring clinicians, patients and families as appropriate and these cases were closed.

At this stage, patients where no VOIs were identified, or where identified VOIs were classified as non‐pathogenic, were grouped together as the remaining undiagnosed patients. Non‐diagnostic reports were generated, and these cases were then investigated using IA‐2.

Follow‐up using IA‐2 coincided with the release of all detected variants by GEL, regardless of original tier. Variants for each case were ranked according to their Exomiser score. Exomiser is a phenotype‐driven java programme used to prioritise variants by estimating their likelihood of causing a certain phenotype, incorporating known phenotype–genotype relationships and other functional data (Smedley et al. 2015). Exomiser access was built into the Congenica platform, allowing users to easily view and prioritise variants, but no specific methodology for interpretation was prescribed by GEL.

In IA‐2 the top five Exomiser variants were identified for all remaining patients, and characteristics of these variants were represented by five key meta‐predictors, manually compiled from online sources. The purpose, application and sources of the meta‐predictors can be seen in Table S2. These meta‐predictors were chosen by lead clinical geneticist for IA‐2 (C.M.) as particularly useful for gauging which variants had stronger molecular evidence for pathogenicity (Joynt et al. 2021). A ‘variant first, phenotype second’ approach was taken where molecularly probable variants were related to phenotypes. Variants which were then deemed plausible causes of phenotypic features were selected as VOIs for formal classification at MDT, as in IA‐1. Some IA‐2 classifications were performed externally by accredited laboratories which also employed ACMG/ACGS criteria. Causative variants were validated with Sanger sequencing and reports for confirmed results were generated and shared.

### 2.3 Service Evaluation and Quality Assurance

Service evaluation and subsequent quality assurance exercises were conducted retrospectively. Patient‐specific clinical data was collected for all diagnosed patients from an internal Laboratory Information Management System (LIMS). Diagnostic yields and TATs were established for the overall study and both individual pathways. TAT was defined as calendar days from study enrolment to the issue of a diagnostic report.

Explanations were sought as to why diagnoses established in IA‐2 were initially missed by IA‐1. Specific information on variant mode of inheritance, original tier ranking and the virtual panels applied was considered. For each patient, their confirmed diagnostic gene was searched on the virtual panels applied during IA‐1, using PanelApp, to determine if detection of the causative variant using panels was actually possible.

The diagnostic potential of both approaches was established by quantifying which could have detected more diagnostic variants. Diagnostic potential was defined as the interpretational approach's ability to detect causative variants and is represented as the proportion of known diagnoses detectable by either approach had all cases undergone both analytical approaches.

## 3 Results

### 3.1 NIRMDS Workflow Performance

In total, *n* = 440 RD patients were recruited to the 100,000 Genomes Project from across NI. Initially only Tier 1 and 2 variants were available for interpretation and of the total probands recruited, 17% (*n* = 75/440) had Tier 1 variants, 35.2% (*n* = 155/440) had Tier 2 but no Tier 1 variants and 10% (*n* = 44/440) had Tier 1 and 2 variants. However, 48.2% (*n* = 212/440) had only Tier 3 variants; therefore diagnosis through IA‐1 was impossible as per GEL pipeline design.

**FIGURE 1:**
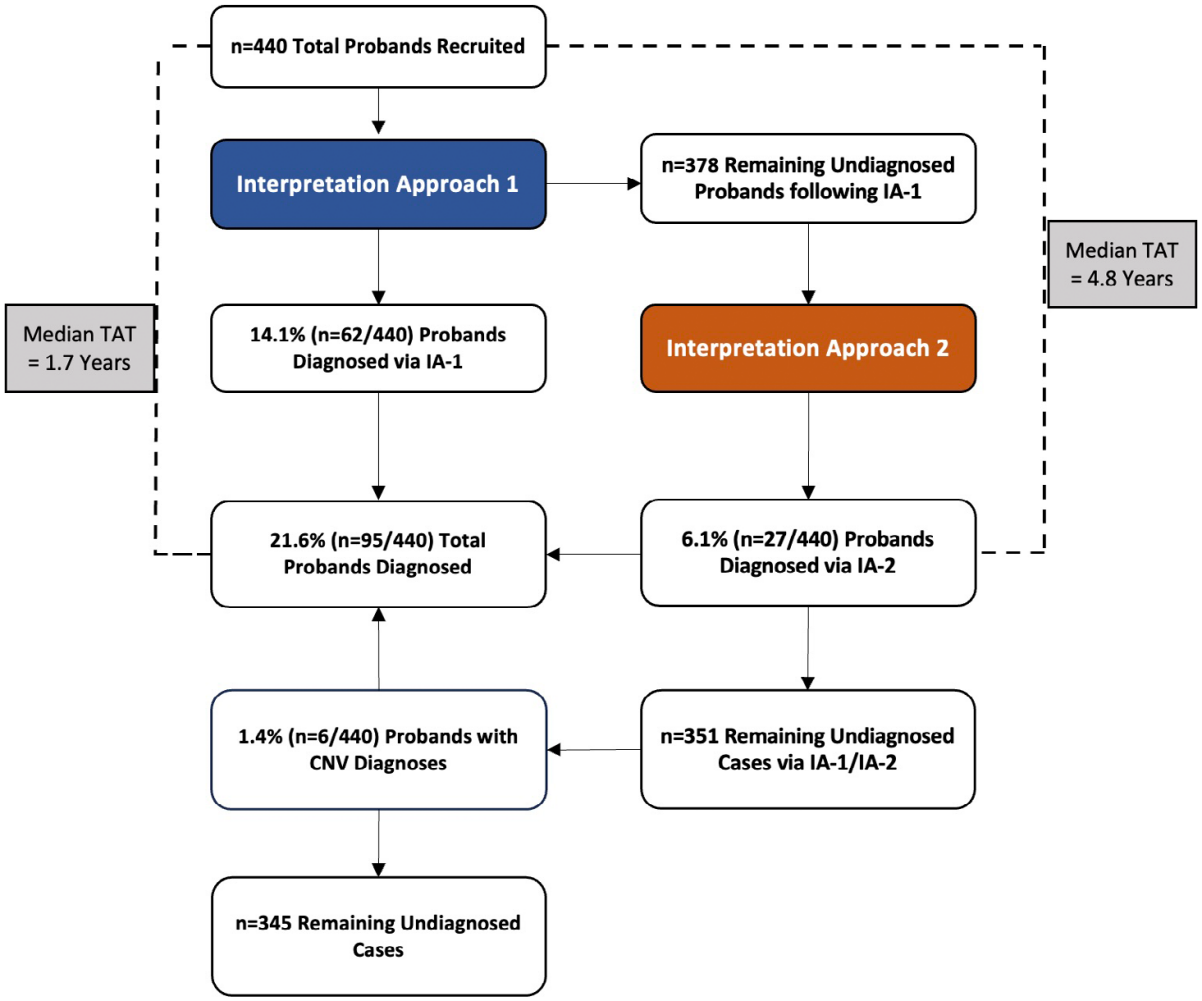
Flowchart summarising diagnoses found by NIRMDS protocol up to July 2025. CNV, copy number variant; TAT, turnaround time.

Overall diagnostic outcomes reported up to July 2025 are summarised in Figure 1 with a breakdown of both approaches.

In total, a yield of 20.2% (*n* = 89/440) was found for patients diagnosed with monogenic disease. Of these, 14.1% (*n* = 62/440) were diagnosed via IA‐1 and 6.1% (*n* = 27/440) via IA‐2. Including diagnoses of chromosomal aberrations, 1.4% (*n* = 6/440), found outside the two‐step approach, the overall yield was 21.6% (*n* = 95/440).

Median TAT overall was 2.1 years, or 778 days (range: 315–2311 days); for IA‐1 it was 1.7 years, or 644.5 days (range: 315–1342 days), and for IA‐2 it was 4.8 years, or 1766.5 days (range: 862–2311 days).

Following IA‐1, *n* = 378 undiagnosed cases remained, of which 7.1% (*n* = 27/378) were diagnosed via the IA‐2 approach. The median time taken from non‐diagnostic IA‐1 report issue to final IA‐2 diagnosis was 3.8 years, or 1397.5 days (range: 862–2311).

Further details on diagnostic single genes and CNVs and variants of unknown significance (VUS) are given in the Supplementary Results.

### 3.2 Diagnoses Missed by Initial Analysis

There were *n* = 27 cases where diagnostic variants were not detected via IA‐1 but found in IA‐2. Three key areas were identified as reasons why diagnoses were not detected, by two experts (S.S. and C.M.). These included; (i) choice of virtual panels applied; (ii) filtering variants by inheritance, and (iii) Clinician's judgement in selecting or rejecting variants (Table 1). Most variants were missed as the diagnostic variant was detected in a gene which was not green on the applied panel(s) at the time of initial analysis—that is, prior to issue of negative report following IA‐1. One case noted to not have the correct panels applied due to incomplete phenotypic information. Case vignettes are provided in the Supplementary Results.

**TABLE 1:** Key areas identified which contribute to diagnostic variants being missed by initial analysis.

	Area contributing to diagnostic variants being missed				
	Applied virtual panel(s)		Filtering by inheritance		Clinician's judgement
Variants originally tiered as:	Tier 3		Tier Null		Tier 1/2
Variants accessible in initial analysis:	N		N		Y
Variant missed because:	Diagnostic variant detected in gene not classed as green on applied virtual panel(s)		Typical mode of inheritance associated with diagnostic syndrome not observed in patient		Diagnostic variant not selected as VOI by referring clinician for classification
Cases affected:	23		2		2
Type of cases affected:	Diagnostic gene not green on applied panel(s) at time of diagnosis – Insufficient evidence available to associate gene with phenotypic outcome	Incorrect panel(s) applied – Potentially due to incomplete phenotypic information	X‐Linked disease in females	Autosomal Dominant inheritance from a seemingly unaffected parent – Potentially due to incomplete penetrance or mosaicism	Diagnostic variant has known phenotypic outcomes which referring clinician does not think aligns with patient's presentation, or thinks other variants align better
Observed examples from audit:	23/27 (85.2%) 2NW‐Diagnosed cases had a diagnostic variant which was not green on applied panel(s) at time of initial analysis*	Proband B001^a^ had incorrect panel(s) applied due to incomplete phenotypic information	Proband B065^b^ is a female affected by an X‐linked disease	Proband B059^c^ has an autosomal dominant diagnostic variant which was paternally inherited. Father displays 30% mosaicism	2/27 (7.4%) of 2NW‐Diagnosed cases had a Tier 1/2 variant. Logically can infer these missed due to clinician's dismissal

*Note:* ^a–c^Individual case vignettes available in Supporting Information.

* Gene was considered not green at time of initial analysis if it was classified as green after non‐diagnostic 1PW report date.

### 3.3 Diagnostic Potential of Interpretational Approaches

The diagnostic potentials of both approaches were calculated with exact 95% confidence intervals (CI). Diagnostic potential of IA‐1 was found to be 69.7% (*n* = 62/89) (95% CI = 59–79), understood as the number of actual diagnoses found via IA‐1.

The diagnostic potential of IA‐2 was estimated as 89.9% (*n* = 80/89) (95% CI = 82–95) of diagnoses that could have been found by secondary analysis. This is understood as *n* = 27 cases actually diagnosed by this approach, plus the number of IA‐1 diagnosed cases which could have been diagnosed if the IA‐2 approach had been applied from the outset. Of the *n* = 62 cases diagnosed via IA‐1, *n* = 53 had their diagnostic variant ranked in the top five Exomiser variants for that patient.

The proportion of cases detectable via IA‐2 was found significantly increased compared to IA‐1, *χ* ^2^ (1, *N* = 89) = 11.28, *p* = 0.0008. Therefore, IA‐2 may be considered more effective in detecting pathogenic variants.

## 4 Discussion

We report a diagnostic yield of 20.2% of monogenic disease within the Northern Ireland cohort recruited to the 100,000 Genomes Project and outline our two‐step interpretation approach for VOI selection. 14.1% were diagnosed via the initial workflow (IA‐1) within a median TAT of 644.5 days and 6.1% were diagnosed via a secondary workflow (IA‐2) within a median TAT of 1766.5 days. Diagnostic variants were most commonly missed in IA‐1 as they were within non‐green genes on applied panels at the time of analysis, meaning that they have a moderate to low degree of evidence supporting the gene‐disease relationship.

The panel‐based analysis used in IA‐1 may be limited in the diagnoses it can access compared with the Exomiser analysis used in IA‐2, but it is a critical tool in alleviating interpretational burdens placed on staff. IA‐1 was employed to rapidly detect the most obvious diagnoses first so diagnostic results could be returned to patients quickly, freeing time for the cases which required a more in‐depth approach. IA‐2 was found more effective in prioritising variants but was also more time‐consuming and labour‐intensive. The diagnostic potential of IA‐2 demonstrates its validity as a follow‐up step which can reliably find missed diagnoses in a smaller subset of patients, making the analysis more efficient overall. It is important to note that we can only retrospectively quantify how many diagnostic variants were ranked in the top five by Exomiser. This does not consider if the clinician would correctly identify VOIs, which requires a sufficient level of knowledge and experience in variant interpretation.

Similar outcomes have been reported for other regional cohorts of the 100,000 Genomes Project in England and Scotland (100,000 Genomes Project Pilot Investigators, et al. 2021; Hocking et al. 2023). The pilot study, based in England, saw a 14.8% yield from panel‐based analysis and an additional 6.1% yield using Exomiser where both approaches were conducted simultaneously for all cases. The Scottish study took a similar two‐step approach to ours with an initial panel analysis with an 18.3% yield and subsequent Exomiser analysis for remaining undiagnosed cases, providing a yield of 4.1%. These studies did not specify how VOIs were selected for MDT classification, so we cannot determine exact reasons for differences in yield, although they are small. However, variation between regions aligning with their local demographics and genetic compositions is expected. Also, the 100,000 Genomes Project had heterogeneous inclusion criteria, recruiting patients affected by a broad spectrum of phenotypic presentations and potential underlying genetic causes (Devereau 2018). Some centres may have been more selective with recruitment. Our diagnostic yield was likely to be reduced as recruits had previously been exposed to extensive single gene/small panel testing before WGS, and yields are typically higher when WGS is a first‐line test.

The TATs were not reported in either of the aforementioned studies, which is common for most NGS publications. Moreover, the definition of TAT in diagnostics is not standardised in the literature. However, reducing TATs is central to shortening the odyssey in RD diagnosis, to alleviate patient anxiety or in some circumstances, give access to specific therapies or care (Yu et al. 2023). NHS England has stated that the TAT for standard RD NGS testing should be around 12‐weeks (East Genomics, n.d.; Manchester University. NHS Foundation Trust, n.d.). This refers to the time taken to return a result, not the time to receive a diagnosis which is more clinically meaningful. Many WGS will have inconclusive results, requiring further analysis. All cases are unique and a typical reanalysis timeframe cannot be prescribed as it cannot be predicted if and when relevant evidence might be published or key phenotypic features might develop in a patient.

Reanalysis of inconclusive NGS cases has increased yields by 3%–15% in various reports representing different disease cohorts (Moon and Seo 2025). The ACMG provides general reanalysis guidelines (Deignan et al. 2019) recommending revisiting undiagnosed cases at a later date to increase yields. These guidelines do not specifically consider variation in time consumed by interpretation approaches. During IA‐2, manual collection of key meta‐predictors was slow and labour‐intensive but was the only viable approach at the time. However, in today's landscape, advancements in artificial intelligence (AI) and machine learning can only improve this, with many modern tools automatically aggregating meta‐variables from multiple sources, reducing time and effort required. Similarly, emerging AI‐powered support tools enhance our ability to identify VOIs, making the overall process more efficient and scalable (Abdelwahab and Torkamaneh 2025).

The yield presented reflects only diagnoses reported at cut‐off of July 2025. Genomic interpretation in a live clinical environment is a dynamic and ongoing process. Beyond this cut‐off, more diagnoses will have been and will continue to be found through reanalysis or wider research partnerships. GEL provides access to research networks enabling professionals investigating similar cases to collaborate and find diagnoses. Through phenotype‐specific Genomics England Clinical Interpretation Partnerships (GeCIPs), a rare cause of Hereditary Haemorrhagic Telangiectasia was identified in NI patients. The diagnosis was made in three members of a family based on identification of a novel variant in *GDF2* (Balachandar et al. 2022). It is noteworthy that reported yields generally focus on outcomes for probands alone, and don't reflect additional people who have received a diagnosis or other clinically relevant findings because they were related to a proband under investigation.

Ultimately, these regional 100,000 Genomes sub‐studies and the wider NGS literature demonstrate that a single analysis approach cannot detect all diagnoses. This suggests that balance between bioinformatic automation and in‐depth expert consideration, allowing for case revisiting, is desirable. While many WGS studies present their results, our study has unique strength in its focus on how clinical practice parallels diagnostic odysseys. In 2022, the NI RD Implementation Group (NIRDIG) published an action plan outlining ‘Achieving diagnosis faster’, (Northern Ireland Rare Disease Implementation Group (NIRDIG) 2022) as a key goal for improving patient lives. Innovation in handling genomic data promotes more rapid diagnosis and we have identified pre‐classification VOI selection as a target area for improvement.

The two‐step interpretation approach was designed to promote collaboration between clinicians and scientists for improvement of patient care. Most WGS clinical settings place staff in specific roles where clinical scientists lead on VOI selection, interpretation and classification, and clinicians provide dysmorphology expertise and gather phenotypic information about the proband, which scientists use to direct the analysis. Our approach is unusual as it emphasises the clinician's role in interpretation, particularly in IA‐2 where a clinical geneticist investigated the molecular basis of pathogenicity for variants. In most NHS genetics services, clinicians are not expected to have an extensive knowledge of molecular genetics and variant interpretation. However, we argue that modern clinical geneticists should be empowered to actively engage with interpretation to support scientists, improve outcomes for their patients, and to simply better understand their daily practice. Unclassified variants, and variants of unknown significance, exist on a spectrum of diagnostic plausibility, and an understanding of meta‐predictors and ACGM/ACGS classification will allow a geneticist to determine which are worthy of further investigation and which can be comfortably dismissed (Joynt et al. 2021).

As the data collection and analysis was conducted retrospectively, care should be taken when interpreting the outcomes. Retrospective data can be inconsistent or incomplete (Talari and Goyal 2020). This in particular applies to the comparison of diagnostic potential of both approaches, as this requires the assumption that all diagnoses would be found using IA‐2 provided they were ranked in the Exomiser top five. This comparison specifically refers to whether finding diagnoses with the approach was possible, not if diagnoses would be found. Therefore, 95% CIs are given with diagnostic potential values to account for this level of uncertainty. Going forward, an ongoing centralised approach to clinical record management would be preferable, especially for future quality reviews.

## 5 Conclusion

The utility of WGS in RD is well‐established and clinical service providers should direct focus to optimising internal protocols to balance yields with staff burden. The virtual gene panels employed by GEL are useful for initially detecting ‘low‐hanging fruit’ diagnoses. However, phenotype‐based panels under‐utilise the full potential of WGS. It is crucial that service providers avoid an exclusively phenotype‐first approach and encourage clinical genetics staff to also consider what is molecularly possible and relate this back to the patient phenotype. As NGS becomes more prevalent, all staff have a role to play, including clinicians. True multidisciplinary collaboration, supported by modern computational tools, allows for an iterative, flexible approach to RD diagnosis, thereby tackling the diagnostic odyssey one case at a time.

## Author Contributions

S.S. and F.M. wrote the manuscript. The clinical research study was performed by S.S., C.M., S.M., A.J.M., S.H., C.H., R.A., K.K. and C.F. All authors approved the final version of the manuscript for submission.

## Funding

Department for the Economy, Northern Ireland; Medical Research Council; Northern Ireland Executive.

## Ethics Statement

Participants gave informed consent when agreeing to participation in the 100,000 Genomes Project. Consent was also provided for any subsequent work related to service provision as addresssed within the index study.

## Conflicts of Interest

The authors declare no conflicts of interest.

## Supporting information


**Figure S1:** Traffic light system to classify genes by colour within GEL virtual panels, used to determine if gene recommended to be used in analysis or not (Nguengang Wakap et al. 2020).
**Table S1:** Tiers which detected variants placed into by GEL panel‐based analysis to prioritise variants most likely to be causative (Wu et al. 2020).
**Table S2:** Meta‐variables collected for the top 5 Exomiser variants which were used to selected VOIs for ACMG/ACGS classification.

## Data Availability

The data that support the findings of this study are available from the corresponding author upon reasonable request.
