# Supplementary material for: A Novel Approach to Interrogating Whole Genome Sequencing Data to Optimise Clinical Utility
Source: Mol Genet Genomic Med. 2026 Jun 11;14(6):e70248. doi: 10.1002/mgg3.70248 (PMC13260672; doi:10.1002/mgg3.70248)
Supplement: Supplementary file 1 — Figure S1: Traffic light system to classify genes by colour within GEL virtual panels, used to determine if gene recommended to be used in analysis or not (Nguengang Wakap et al. 2020). Table S1: Tiers which detected variants placed into by GEL panel‐based analysis to prioritise variants most likely to be causative (Wu et al. 2020). Table S2: Meta‐variables collected for the top 5 Exomiser variants which were used to selected VOIs for ACMG/ACGS classification. [file MGG3-14-e70248-s001.docx]

A Novel Approach to Interrogating Whole Genome Sequencing Data to Optimise Clinical Utility - Supplementary Material.

1. Supplementary Methods:


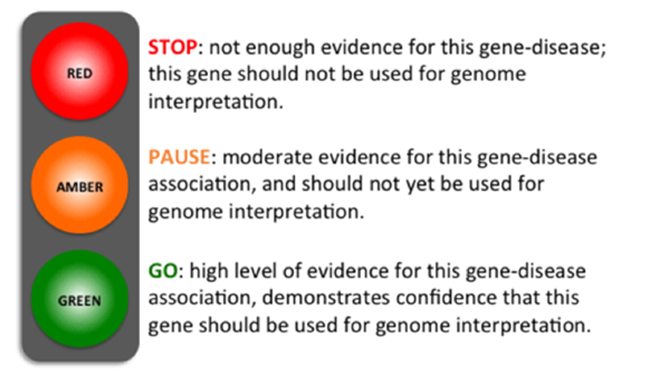


Supplementary figure 1: Traffic light system to classify genes by colour within GEL virtual panels, used to determine if gene recommended to be used in analysis or not.^1^

| Tier | Explanation |
| --- | --- |
| Tier 1 | High Impact Variants e.g. Loss-Of-Function or *de novo* (With confirmed parentage) Moderate Impact Variants E.g. Missense detected in Green Genes on applied virtual panel(s) with sufficient evidence linking the phenotype. |
| Tier 2 | Moderate Impact Variants E.g. Missense detected in Green Genes on applied virtual panel(s) with sufficient evidence linking the phenotype. |
| Tier 3 | Potentially Disease-Causing Variants (Including High and Moderate Impact) detected in genes which are not green on applied panel(s). |
| Tier Null | Variant observed in healthy population or does not follow appropriated mode of inheritance. |

Supplementary table 1: Tiers which detected variants placed into by GEL panel-based analysis to prioritise variants most likely to be causative.^2^

| Meta-Predictor | Description | Application |
| --- | --- | --- |
| OMIM Morbidity^3^ | Online Mendelian Inheritance in Man database collates information about Mendelian disorders and associated genes. An OMIM Morbid gene has an evidence-based link to human disease. | Variants found in OMIM Morbid genes considered more likely to be causative due to significant levels of available evidence associating gene with disease. |
| GnomAD Allele Count^4^ | Number of times variant observed in Genome Aggregation Database - A publicly available source of aggregated global genome and exome sequencing data. Variants found in unaffected populations unlikely to be pathogenic. | Variants absent in unaffected/healthy populations considered more likely to be causative. |
| REVEL Score^5^ | Predicts pathogenicity of missense variants using a combination of data from 13 tools*. Score can range from 0 to 1 and higher scores indicate increased likelihood of pathogenicity. | For Missense Variants - Score >0.7 considered significant as evidence of pathogenicity. |
| Z-Score^6^ | Quantifies missense constraint of gene region. Positive scores indicate more constraint and negative scores less. Highly constrained regions are less tolerant to missense variation meaning variation more likely to be pathogenic. | A Missense Variant occurring in a gene region with a Score >3.09 considered significant as evidence for pathogenicity. |
| pLi^7^ | Quantifies tolerance of a gene region to truncating variation. Score ranges from 0 to 1 and scores closer to 1 indicate low tolerance, meaning truncating variation more likely to be pathogenic. | A Loss-Of-Function/Protein Truncating Variant occurring in a gene region with a Score >0.9 considered significant as evidence for pathogenicity. |

Supplementary table 2: Meta-variables collected for the top 5 Exomiser variants which were used to selected VOIs for ACMG/ACGS classification.

*13 Tools incorporated in REVEL Score: MutPred, FATHMM v2.3, VEST 3.0, PolyPhen-2, SIFT, PROVEAN, MutationAssessor, MutationTaster, LRT, GERP++, SiPhy, phyloP, and phastCons.

1. Supplementary Results:

- Vignette 1 – B001

Patient enrolled in 100,000 Genomes Project with testicular enlargement, global developmental delay, autism, learning difficulties, overweight and with coarse features. Based on this phenotypic information provided, the Beckwith-Wiedemann Syndrome (BW) and other congenital overgrowth disorders v1.42 and Intellectual disability v2.597 virtual panels were applied to associated variant data. However, the key phenotypic detail – hypothyroidism, was omitted, and so the congenital hypothyroidism panel was not applied and proband remained undiagnosed at end of 1PW.

On reanalysis in 2NW, a hemizygous maternally inherited variant was identified in *IGSF1*, which was in fact a green gene on the hypothyroidism panel prior to proband’s enrolment in the study. The variant was deemed likely pathogenic and consistent with the features of *IGSF1-Deficiency Syndrome*. Moreover, *IGSF1* was, and still is, a red gene on the Intellectual Disability Panel, meaning that variants within that gene have insufficient evidence of being the cause of intellectual disability and should not be considered for interpretation.

Often, hypothyroidism can be the root cause of intellectual disability^8^, and applying the intellectual disability panel meant that the GEL panel-based bioinformatic pipelines were searching for a downstream consequence rather that the underlying issue. It is possible that hypothyroidism was not submitted as a phenotypic term as an error, or it had simply not been diagnosed in patient yet.

- Vignette 2 – B065

Female patient who enrolled in 100,000 Genomes Project presenting with microcephaly, severe developmental delay, seizures, arched eyebrows, anteverted nares, a small chin, flexed elbows with extended legs and holoprosencephaly.

Patient diagnosed in 2NW with *SMC1A-Related Neurodevelopmental Disorder* caused by a heterozygous *de novo* variant within *SMC1A* which was classified as pathogenic. This variant, despite being causative and also green on the applied virtual panels, was filtered out as Tier Null as it did not demonstrate a typical mode of inheritance. *SMC1A* is found on the X chromosome and X-linked disease is incredibly rare in females.^9^

X-linked disease in females is rare due to compensation provided by their second X chromosome. However, it is possible, and often this compensatory effect creates a milder phenotype than is seen in their male counterparts. A mild phenotype may result in patients and their families being less likely to pursue medical investigation as the condition may have less significant impact on their health. This can lead to fewer reported cases and therefore limited research for comparison to facilitate diagnosis in others.

- Vignette 3 – B059

Patient enrolled in 100,000 Genomes Project with low-set ears, abnormality of finger, mild intellectual disability, ventricular septal defect, morphological abnormality of the central nervous system, delayed gross motor development, Arnold Chiari type 1 malformation, moderately short stature, short metacarpal, 3-5 finger syndactyly, delayed fine motor development, mild global developmental delay.

Diagnostic variant found in green gene on applied panels but was not flagged in 1PW. Diagnosis achieved in 2NW with a heterozygous variant in *SALL1* gene which was classified as likely pathogenic and consistent with *Townes-Brocks Syndrome*.

The variant was filtered out as Tier Null for displaying an unusual mode of inheritance, as it was paternally inherited, and father was recorded as unaffected. The bioinformatic pipelines therefore assumed this variant was highly unlikely to be diagnostic as it seemingly did not produce features of syndrome in father. However, the father actually displayed ~30% somatic mosaicism which meant that variant effects were not noted before.

Mosaicism across many genetic diseases can result in a milder phenotype^10^ in a patient than is usually seen. It has also been noted in the literature that *Townes-Brocks Syndrome* specifically can have a milder presentation in a mosaic patient.^11^

[1PW = Initial Positive Workflow; 2NW = Follow-up Negative Workflow]

REFERENCES

1. Sarah Leigh, et al. Genomics England Quality Management System – PanelApp Handbook. GUI-BIO-004. v35. 10/11/2022. Pg 13, 8.5 Understanding Gene Ratings on a Version 1+ Gene Panel.
2. Genomics England. Genomics England Research Environment User Guide. Rare disease tiering. [https://re-docs.genomicsengland.co.uk/tiering/#rare-disease-tiering](#rare-disease-tiering). Accessed 11/03/2025.
3. Online Mendelian Inheritance in Man, OMIM^®^. McKusick-Nathans Institute of Genetic Medicine, Johns Hopkins University (Baltimore, MD), {date}. World Wide Web URL: https://omim.org/
4. Chen, S.*, Francioli, L. C.*, Goodrich, J. K, et al. A genomic mutational constraint map using variation in 76,156 human genomes. *Nature*. 625, 92-100 (2024). https://doi.org/10.1038/s41586-023-06045-0 PMID: 38057664
5. Ioannidis NM, Rothstein JH, Pejaver V, et al. REVEL: An Ensemble Method for Predicting the Pathogenicity of Rare Missense Variants. Am J Hum Genet. 2016 Oct 6;99(4):877-885. doi: 10.1016/j.ajhg.2016.08.016. Epub 2016 Sep 22. PMID: 27666373; PMCID: PMC5065685.
6. Betancur C, Buxbaum JD. Gene constraint and genotype-phenotype correlations in neurodevelopmental disorders. Curr Opin Genet Dev. 2020 Dec;65:69-75. doi:10.1016/j.gde.2020.05.036. Epub 2020 Jun 26. PMID: 32599522; PMCID: PMC10340126.
7. Lek, M et al. “Analysis of Protein?Coding Genetic Variation in 60,706 Humans.” *Nature.* 536.7616 (2016): 285–291.
8. Uchida K, Suzuki M. Congenital Hypothyroidism and Brain Development: Association With Other Psychiatric Disorders. Front Neurosci. 2021 Dec 9;15:772382. doi: 10.3389/fnins.2021.772382. PMID: 34955723; PMCID: PMC8695682.
9. Migeon, B.R. X-linked diseases: susceptible females. *Genet Med* **22**, 1156–1174 (2020). <https://doi.org/10.1038/s41436-020-0779-4>
10. Rebecca Truty, Susan Rojahn, Karen Ouyang, Curtis Kautzer, Michael Kennemer, Daniel Pineda-Alvarez, Britt Johnson, Amanda Stafford, Lina Basel-Salmon, Sulagna Saitta, Anne Slavotinek, Settara C. Chandrasekharappa, Carlos Jose Suarez, Leslie Burnett, Robert L. Nussbaum, Swaroop Aradhya, Patterns of mosaicism for sequence and copy-number variants discovered through clinical deep sequencing of disease-related genes in one million individuals, The American Journal of Human Genetics, Volume 110, Issue 4, 2023, Pages 551-564, ISSN 0002-9297,https://doi.org/10.1016/j.ajhg.2023.02.013.
11. Devriendt, K., Fryns, J. P., Lemmens, F., Kohlhase, J., & Liebers, M. (2002). Somatic mosaicism and variable expression of Townes-Brocks syndrome. *American journal of medical genetics*, *111*(2), 230–231. https://doi.org/10.1002/ajmg.10485
